# A Heuristic Solution of the Identifiability Problem of the Age-Period-Cohort Analysis of Cancer Occurrence: Lung Cancer Example

**DOI:** 10.1371/journal.pone.0034362

**Published:** 2012-04-04

**Authors:** Tengiz Mdzinarishvili, Simon Sherman

**Affiliations:** Eppley Institute, University of Nebraska Medical Center, Omaha, Nebraska, United States of America; Sapienza University of Rome, Italy

## Abstract

**Background:**

The Age–Period–Cohort (APC) analysis is aimed at estimating the following effects on disease incidence: (i) the age of the subject at the time of disease diagnosis; (ii) the time period, when the disease occurred; and (iii) the date of birth of the subject. These effects can help in evaluating the biological events leading to the disease, in estimating the influence of distinct risk factors on disease occurrence, and in the development of new strategies for disease prevention and treatment.

**Methodology/Principal Findings:**

We developed a novel approach for estimating the APC effects on disease incidence rates in the frame of the Log-Linear Age-Period-Cohort (LLAPC) model. Since the APC effects are linearly interdependent and cannot be uniquely estimated, solving this identifiability problem requires setting four redundant parameters within a set of unknown parameters. By setting three parameters (one of the time-period and the birth-cohort effects and the corresponding age effect) to zero, we reduced this problem to the problem of determining one redundant parameter and, used as such, the effect of the time-period adjacent to the anchored time period. By varying this identification parameter, a family of estimates of the APC effects can be obtained. Using a heuristic assumption that the differences between the adjacent birth-cohort effects are small, we developed a numerical method for determining the optimal value of the identification parameter, by which a unique set of all APC effects is determined and the identifiability problem is solved.

**Conclusions/Significance:**

We tested this approach while estimating the APC effects on lung cancer occurrence in white men and women using the SEER data, collected during 1975–2004. We showed that the LLAPC models with the corresponding unique sets of the APC effects estimated by the proposed approach fit very well with the observational data.

## Introduction

For more than 50 years, the importance of accurate accounting for the Age–Period–Cohort (APC) effects has been well recognized by epidemiologists and mathematicians in disease incidence and mortality studies. In such studies, the incidence rate is defined as a ratio of the number of events divided by the total person-years experience. It is assumed that the numerator of this ratio has a Poisson distribution and the standard errors (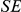) of the incidence rate are calculated by the ratio of the squared root of the number of events divided by the total person-years [1]. Often, it is also assumed that the logarithm of the incidence rate can be modeled as a linear function of specified regressors: the APC effects. Such models of the incidence rates belong to the so-called generalized linear models [2]. In particular, in the Log-Linear Age-Period-Cohort (LLAPC) model, the observed variable is the logarithm of the incidence rate, which is approximated by the sum of the APC effects [2]. The problem is figuring out how to estimate these effects from the observed incidence rates.

### APC analysis

In this work, using the long-term observational data, we determine the APC effects in the frame of the LLAPC model [2]. By definition [1], the crude incidence rate for the given age, time-period (TP) and birth-cohort (BC) intervals, is a ratio of the number of cancer occurrences, 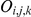, divided by the total person-years at risk, 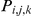:
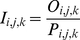
(1)where the age intervals are indexed as 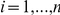; the time periods of cancer occurrences as 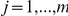; the birth cohorts of cancer occurrences as 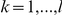; and 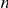, 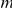 and 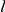 are numbers of the age intervals, time periods, and birth cohorts, correspondingly.

Let us consider that the temporal intervals, indexed by 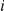, 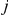 and 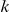, have the same size (for instance, five-year long intervals that are usually used in the APC studies). In this case, these indexes and the 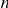, 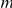 and 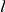 numbers are related in the following way [2]:
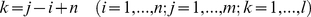
(2)and 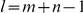. It should be noted that, according to (2), index 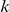 is uniquely defined by indexes, 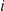 and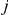. Therefore in (1), index 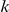
can be omitted, while keeping in mind that incidence rates are also dependent on the BC effects.

The LLAPC model is usually presented by the following system of conditional equations:
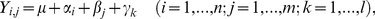
(3)and
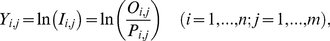
(4)where 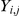 is a logarithm of the observed incidence rate, 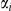 denotes the age effect, 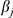 - the TP effect, 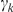 - the BC effect, and the constant term, 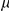, is the intercept [2]. In this model, weights for the observed data, 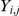, are chosen to be inversely proportional to their sampling variances,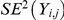:
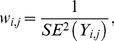
(5)where
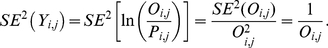
(6)Formula (6) is obtained under the assumption that the numbers of cancer occurrences in each group are independent random variables characterized by a Poisson distribution. It is also assumed that the variances of the incidence rates, 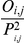, are entirely due to variations in the small number of cancer occurrences, 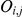, compared to the total person-years at risk, 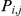, [3]. From (5) and (6) it follows that:
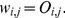
(7)The APC problem is to determine from the system of 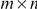 conditional equations (3) with weights (7) the following: (i) the 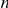 estimates of the age effects, 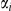; (ii) the 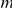 estimates of the TP effects,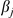; (iii) the 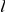 estimates of the BC effects, 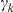; and (iv) the intercept, 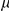. Additional constraints on the parameters must be made to obtain a solution. One approach is to set three effects (one of the TP effects, 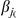, one of the BC effects, 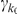, and the corresponding Age effect, 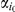, where 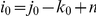) to zero and then to use these settings as the reference levels. Another approach is to set the sums of these effects to zero [2]. In the present work, we use the first approach.

From the aforementioned settings and from (1–7), it follows that:

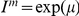
 presents the modeled incidence rate of getting the cancer, when the anchored parameters are: 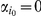, 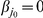, and 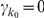.

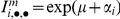
 presents the modeled Age-specific incidence rate of getting the cancer in a given age interval 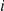, when TP and BC effects are absent.

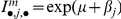
 presents the modeled TP-specific incidence rate of getting the cancer for a given TP interval, 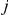, when Age and BC effects are absent.

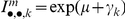
 presents the modeled BC-specific incidence rate of getting the cancer for a BC interval, 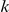, when Age and TP effects are absent.

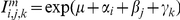
 presents the modeled incidence rate of getting a particular type of cancer in a given Age interval, 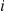, a TP interval, 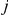, and a BC interval, 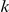, when all of these effects are present.

In 2), 3) and 4), the Age effects,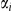, the TP effects,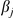, and the BC effects, 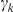, can be presented as logarithms of the incidence rate ratios: 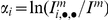, 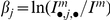, and 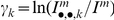, correspondingly. Thus, the 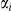,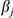, and 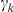 parameters are dimensionless and their variations (with respect to the corresponding successive Age, TP and BC intervals) indicate the temporal trends of these effects.

### Identifiability problem

The system (3) cannot be solved directly by methods of multiple linear regressions due to the fact that the design matrix of the system (3) of the LLAPC is rank deficient. (This fact can be directly checked in practice, for example, using MATLAB function, *rank*). This is because the APC effects are linearly interrelated. Consequently, these effects cannot be uniquely and simultaneously estimated (multiple estimators of these parameters provide similar solutions). Mathematically, this problem falls into a category of the *identifiability* problems that, in turn, are a special subclass of a more general class of the *ill-posed* or *incorrectly-posed* mathematical problems. Solving the identifiability problem, in particular, and the ill-posed problems, in general, requires the use of additional assumptions and/or *a priori* knowledge regarding their solutions [4].

Approaches that have been used in the APC analysis to solve the identifiability problem are reviewed in several papers (see, for example, [2], [5], [6] and references therein). In these approaches, either three effects (one of the TP effects, one of the BP effects, and the corresponding Age effect) are set to zero and used as reference levels or the sums of these effects are equated to zero. However, these settings are still insufficient for solving the identifiability problem [2] and required the use of additional constraints on a set of the parameter estimates to be determined. Although a variety of additional constraints and the utility of estimable functions (that are invariant for any particular set of model parameters) have already been proposed, the identifiability problem still remains largely unsolved [2], [5], [6].

In this work, we extended the well-known approach used in the APC analysis for solving the identifiability problem [2], [3], [7], [8], where four redundant parameters within a set of the unknown parameters to be determined are equated to zero. In our approach, we fixed (set to zero) only three redundant parameters and used them as reference levels. In contrast to the “traditional” approaches, where all four parameters are equated to zero, we determined an optimal value of the forth parameter using an additional heuristic assumption (see below). We used an effect of the time period adjacent to the anchored time period as such a parameter. We have shown that by varying this parameter from −∞ to ∞, all possible solutions of the APC problem can be obtained. To our best knowledge, such a general solution of the APC problem (a complete family of estimates of the APC effects) which depends only on the one “identifiability” parameter is given for the first time in the present work.

### A heuristic assumption

To get an optimal value of the identification parameter, we used a heuristic assumption that the effects of the adjacent cohorts are close. This assumption is motivated by the fact that the multi-year adjacent birth-cohorts are overlapping in time intervals. Using this assumption, we developed a numerical method for determining the optimal value of the identification parameter. With the optimal value of this parameter, a unique set of the APC effects can be determined and thus the identifiability problem is overcome. The method for obtaining the optimal value of the identifiability parameter proposed in this work enables one to obtain a distinct solution(s) of the APC identifiability problem depending on a *priori* assumption(s).

### Proof-of-concept

We tested the proposed numerical method while estimating the APC effects on lung cancer (LC) incidence rates in white men and women, using data collected in the SEER 9 database during 1975–2004.

## Materials and Methods

### Data preparation

To test the proposed approach, we used the SEER databases that include the number of occurrences of different types of cancer and information on the population at risk obtained from the U.S. Census Bureau. In our study, data on LC occurrence in white men and women collected in SEER 9 during 1975–2004 [9] were utilized. We used data from the nine registries rather than data from the currently available 17 registries, because the longitudinal nature of our study required utilization of data dating back three decades when there were only nine registries.

From SEER 9, we extracted the first primary, microscopically confirmed LC cases stratified by gender and race. The number of the LC occurrences in white men and women and the corresponding person-years at risk extracted from the SEER 9 were grouped in six five-year cross-sectional TP groups: 1975–79, … , 2000–04; 18 five-year age groups: 17 groups, ranging from 0 to 84 years, and the 18th group including all cases for the ages 85+; and 17 BC groups corresponding to the birth year groups of 1890–94, …, 1970–74. In our study, we used only 12 five-year Age groups from 30–34 years up to 85+, because the observed numbers of the LC cancer occurrences in younger ages were insignificant. The grouped data, tabulated by the age and time-period indexes, are presented in Tables 1, 2, 3, 4.

**Table 1:** Numbers of LC occurrences,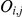 (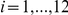;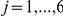), in white men.

	Time-period, *j*					
Age, *i*	1	2	3	4	5	6
**1**	62	56	66	56	47	34
**2**	186	199	189	170	157	127
**3**	447	462	502	436	427	391
**4**	1289	1042	1019	993	920	874
**5**	2522	2260	1971	1754	1723	1646
**6**	3701	3988	3554	2794	2679	2548
**7**	4691	5150	5242	4505	3667	3455
**8**	4629	5581	5828	5927	4891	3903
**9**	3825	4742	5266	5320	5098	4495
**10**	2428	3097	3641	3977	4026	3970
**11**	1112	1414	1735	1907	2160	2233
**12**	430	611	688	806	882	1046

**Table 2:** Numbers of LC occurrences,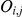 (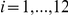;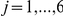), in white women.

	Time-period, *j*					
Age, *i*	1	2	3	4	5	6
**1**	54	43	48	61	49	47
**2**	137	163	148	146	191	130
**3**	338	376	363	362	354	438
**4**	714	655	752	798	817	841
**5**	1157	1340	1230	1342	1406	1371
**6**	1642	1997	2099	2013	2068	2171
**7**	1793	2438	2906	2818	2723	2774
**8**	1563	2557	3212	3743	3776	3115
**9**	1143	1984	2900	3610	4044	3753
**10**	696	1228	2027	2774	3340	3496
**11**	334	614	959	1408	1925	2215
**12**	162	310	460	582	857	1078

**Table 3:** Person-years at risk, 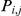
(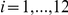;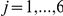), in white men.

	Time-period, *j*					
Age, *i*	1	2	3	4	5	6
**1**	3284057	3855379	4218571	4424306	4174881	3887514
**2**	2598673	3175924	3771661	4219556	4424293	4096518
**3**	2275776	2512806	3135608	3793720	4153454	4306562
**4**	2325082	2186474	2468035	3068524	3666173	4037804
**5**	2404950	2229637	2105450	2374601	2977762	3582781
**6**	2204543	2211782	2053507	1977980	2245790	2829250
**7**	1821543	1961338	1959708	1865543	1803522	2053431
**8**	1389295	1552676	1684938	1701145	1628237	1576438
**9**	996592	1122555	1250158	1388558	1429569	1377036
**10**	652571	736459	848489	980199	1109274	1144139
**11**	401832	418328	473233	562482	679997	780334
**12**	262164	294780	315600	362588	444092	563766

**Table 4:** Person-years at risk, 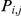 (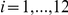;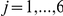), in white women.

	Time-period, *j*					
Age, *i*	1	2	3	4	5	6
**1**	3277344	3828844	4155771	4300824	3981556	3645262
**2**	2599490	3155782	3737364	4147045	4306404	3920423
**3**	2300756	2542175	3160584	3788868	4119406	4229870
**4**	2382884	2223398	2483822	3068156	3669074	4030720
**5**	2530882	2304491	2155724	2419363	3029227	3631242
**6**	2368611	2388806	2169153	2056652	2310912	2903761
**7**	2025933	2179103	2181316	2031330	1933061	2163455
**8**	1731036	1896283	2027322	2033914	1876816	1773427
**9**	1396446	1550817	1682718	1806203	1819431	1685872
**10**	1066964	1197864	1333645	1470545	1599559	1592268
**11**	763277	821648	921957	1044540	1168045	1265313
**12**	586549	734488	842347	975578	1130917	1296494

### Statistical methods and software used

For data presented in Tables 1, 2, 3, 4, the LLAPC model was applied and the corresponding design matrices of the systems of conditional equations for white men and women were obtained. These design matrices were checked for rank deficiencies using the MATLAB function, *rank*. To solve these systems of conditional equations, we applied a novel approach (see below) using the weighted least-square method and utilized the MATLAB function, *regress*. For determining the optimal values of the identification parameters, we used a program developed in-house, *inpar*, and written in MATLAB, Version 7.10.0 (R2010a). Validity of the used LLAPC models for assessing the APC effects in the LC occurrences in white men and women were checked by three diagnostic plots [10]: (i) the normal probability plot of the standardized residuals, (ii) the residuals *vs.* the modeled values plot; and (iii) the observed *vs.* the modeled values plot.

### A solution of the identifiability problem

Let us fix one of the TP effects, 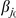, one of the BC effects, 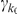, and the corresponding Age effect, 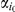, where 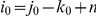 (see (2)). By moving these effects to the left side of the system (3), the number of unknowns in a new system is decreased by three. In practice, these effects are used as reference levels and are usually set to zero.

In such a case, the solution of the APC problem is reduced to determining one parameter – the identification parameter. Let us use the effect, 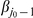 (or 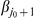) of the TP, adjacent to the anchored TP, 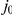, as the identification parameter designated by 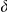. When the exact value of 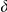 is *a priori* known, the system (3) can be additionally corrected for this effect by moving this parameter to the left side of (3). Then the left sides of the corrected system will be:
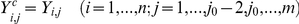


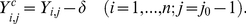
(8)Note, when the exact value of 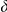 is *a priori* known, the corrected system (3) has the same weights (7) as system (3) and the design matrix of this weighted system does not have a rank deficiency (this can be directly checked by using the MATLAB function, *rank*). For assessing the unknowns in the corrected system (3), a standard weighted least squares method can be used. Thus, estimates of the intercept,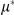, the 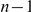 numbers of the Age effects, 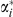, the 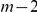 numbers of the estimates of the TP effects, 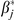, and the 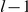 numbers of the estimates of the BC effects, 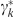, and their confidence intervals (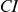) can be obtained. Here and below, asterisks 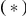
denote estimates or set values of the unknown parameters. It should be noted that, in general, these estimates depend on given values of the four redundant parameters: 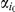, 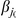, 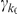 and 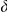.

By varying the identification parameter, 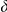, within the interval of its expected variation, a family of estimates of the APC effects can be obtained. In fact, let us suppose the values of the expected variation of the identification parameter lie within an interval, 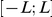, where 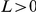. In this interval, let us choose the following net points:
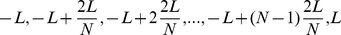
(9)where 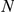
is a natural number bigger than, say, 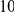, *i.e.* 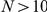. The consequent values of these net points can be used as the variable values of the identification parameter:
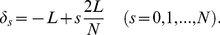
(10)For each 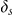
value, one can obtain estimates of the APC effects (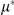, 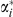, 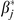, and 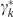) and their 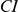
s, as was described previously.

Thus, the corresponding family of estimates of the APC effects can be obtained. Theoretically, by varying 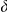 from 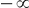 to 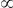, one can obtain all possible estimates of the APC effects (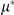, 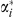, 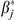, and 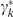) and their 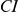
s.

The optimal value of the identification parameter,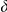, can be determined within the interval of its expected variation using an additional assumption. As such, the heuristic assumption that differences between the effects of the adjacent birth-cohorts are small can be used. This assumption is based on the fact that the multi-year adjacent birth-cohorts are overlapping in time intervals, and the identification of a cohort associated with a particular range for period and age is somehow ambiguous [11]–[13].

Using this heuristic assumption, one can numerically determine the optimal value of the identification parameter by minimizing (with respect to 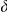) the weighted average of the squared differences between the estimates of the adjacent BC effects, 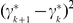. This minimization problem can be formulated as follows:
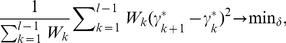
(11)where the weights, 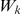, are reciprocals of the variances of the differences between estimates of the adjacent BC effects, 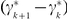. This problem can be solved numerically by getting the net values (10), and calculating for each 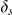 the corresponding weighted average (11). Thus, from these net values, the optimal value,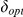, which minimizes this weighted average, can be obtained.

### Assessing model adequacy

To check the goodness of the fit of the modeled values obtained by a multiple linear regression analysis of the observed values, the 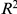 statistic as well as the 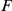 statistic and its 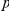 value, are usually used. However, to compute these statistics, the design matrix of the system of the conditional equations, presenting the model under consideration, has to include a column with “1”. Otherwise, the obtained numeric values of these statistics can be incorrect and even erroneous [14], [15]. In our case, the design matrix of the system of the weighted conditional equations of the corrected system (3) with weights (7) does not include the column with “1”. Therefore, for assessing the validity of the results obtained by the proposed approach, we utilized the following diagnostic plots [10]: (i) the normal probability plot of the standardized residuals; (ii) the residuals *vs.* the modeled values plot; and (iii) the observed *vs.* the modeled values plot. Plot (i) allows one to assess the plausibility of the assumption that standardized residuals, 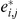
(the observed weighted values, 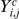 less the modeled weighted values, 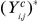, divided by their estimated 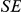), have a normal distribution. If the assumption of normally distributed residuals is correct, the plot should be sufficiently straight. Plot (ii) checks the aptness of the model. When the model is appropriate, the residuals should be randomly distributed around 0, so all, but a very few 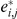 (about 95% of the total number of residuals) should lie between the values of −2 and 2. Plot (iii) should exhibit points located close to the line with a slope of +1 going through the point (0, 0). This plot provides a visual assessment of the effectiveness of the model in making predictions.

## Results

In this section, we present the results of the testing of this approach, while estimating the APC effects on lung cancer (LC) incidence rates in white men and women, using SEER 9 data, collected over a 30-year time period.

### Testing of the approach

The SEER 9 data collected during 1975–2004 for LC in white men and women were used for testing of the proposed approach. In this testing, preparation of the SEER-based data was performed as described in the Materials and Methods section. The obtained number of cancer occurrences and the total person-years at risk for the given age intervals and time periods are presented in Tables 1, 2, 3, 4.

Data presented in Tables 1, 2, 3, 4 were used to obtain the crude incidence rates and their variances. The tabular presentation of the logarithms of these incidence rates is shown in Table 5. In this table, the LC incidence rate data are portioned in to six time periods (1975–79, …, 2000–04 the modeled Age-specific incidence rates,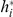), 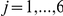; 17 BC groups (1890–94,…,1970–74), 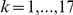; and 12 Age groups (30–34,…,80–84,85+), 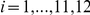. Here, the cross-sectional incidence rates are shown in the columns. The rows of this table show the incidence rates for 12 Age groups. The incidence rates for 17 BC groups (longitudinal data) are presented along the upper-left to lower-right diagonals. The logarithm of the incidence rate of the anchored cell (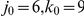) is denoted by a “+” symbol. The problem is to estimate: 12 Age effects (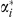); six TP effects (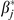); 17 BC effects (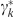); and the intercept (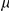). In total, 36 unknown parameters have to be determined from 72 observed values of 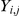 (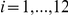;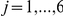).

**Table 5:** Tabular presentation of the logarithms of the observed incidence rates, 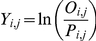 (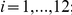 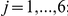 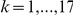), in the frame of the LLAPC model.

	Time-period, *j*						
Age, *i*	1	2	3	4	5	6	Birth-cohort, *k*
**1**	*Y_1,1_*	*Y_1,2_*	*Y_1,3_*	*Y_1,4_*	*Y_1,5_*	*Y_2,6_*	***17***
**2**	*Y_2,1_*	*Y_2,2_*	*Y_2,3_*	*Y_2,4_*	*Y_2,5_*	*Y_2,6_*	***16***
**3**	*Y_3,1_*	*Y_3,2_*	*Y_3,3_*	*Y_3,4_*	*Y_3,5_*	*Y_3,6_*	***15***
**4**	*Y_4,1_*	*Y_4,2_*	*Y_4,3_*	*Y_4,4_*	*Y_4,5_*	*Y_4,6_*	***14***
**5**	*Y_5,1_*	*Y_5,2_*	*Y_5,3_*	*Y_5,4_*	*Y_5,5_*	*Y_5,6_*	***13***
**6**	*Y_6,1_*	*Y_6,2_*	*Y_6,3_*	*Y_6,4_*	*Y_6,5_*	*Y_6,6_*	***12***
**7**	*Y_7,1_*	*Y_7,2_*	*Y_7,3_*	*Y_7,4_*	*Y_7,5_*	*Y_7,6_*	***11***
**8**	*Y_8,1_*	*Y_8,2_*	*Y_8,3_*	*Y_8,4_*	*Y_8,5_*	*Y_8,6_*	***10***
**9**	*Y_9,1_*	*Y_9,2_*	*Y_9,3_*	*Y_9,4_*	*Y_9,5_*	*Y_9,6_^+^*	***9***
**10**	*Y_10,1_*	*Y_10,2_*	*Y_10,3_*	*Y_10,4_*	*Y_10,5_*	*Y_10,6_*	***8***
**11**	*Y_11,1_*	*Y_11,2_*	*Y_11,3_*	*Y_11,4_*	*Y_11,5_*	*Y_11,6_*	***7***
**12**	*Y_12,1_*	*Y_12,2_*	*Y_12,3_*	*Y_12,4_*	*Y_12,1_*	*Y_12,6_*	***6***
	***1***	***2***	***3***	***4***	***5***	***6***	

Using Table 5 and formulas (3) and (7), the design matrices for the LLAPC model of LC in white men and women were built and their rank deficiencies were checked (see Materials and Methods). The obtained rank deficiencies of these design matrices were equal to 4. Therefore, four parameters had to be fixed to determine the APC effects for LC in white men and women by using the corresponding systems of the conditional equations (3) with weights (7). This was done in two steps: (i) by choosing one of the Age effects, one of the TP effects, and one of the BC effects as anchors and setting them to 0; and (ii) by determining the optimal value of the identification parameter – effect of the TP, adjacent to the anchored TP.

To perform the first step, we chose the cell with indexes 9 and 6 (i.e. 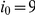 and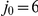) as the anchored cell in Table 5. This means that the Age interval, 70–74, and the TP of 2000–04 (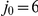) were chosen as the anchors. Since the indexes, 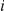, 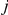 and 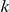 are linearly interrelated by formula (3), the anchored BC index was 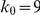. This index corresponds to the BC group of 1925–29. To perform the second step, we chose the TP effect, adjacent to the anchored TP, i.e. 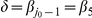. Then, we moved this identification parameter as well as the anchored parameters to the left side of the system (3). For the anchored cell, (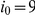,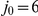, 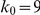), we set the corresponding APC effects to zero and used these effects as the reference levels.

For the obtained conditional systems of equations (8) with weights (7), we built the corresponding design matrices and checked the rank deficiencies of these matrices by using the Matlab function, *rank*. We found that these matrices do not have a rank deficiency and their full ranks were equal to 32. We applied the aforementioned numerical procedure for obtaining 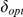 from the net values (11), when 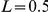 and 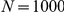.

To determine the optimal value of the identification parameter, 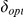, we used our program, *inpar*, and obtained the values of 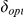
∼0.14 and 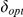
∼0.03, for men and women, correspondingly. These optimal values of the identification parameter were used for estimating the APC effects (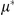, 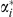, 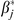, and 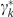), as well as the lower (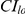) and upper (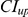) bounds of their 95% confidence intervals for LC in white men and women. For men, the obtained estimates of the intercept,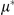, and its 95% 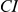 with the lower (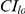) and upper (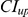) bounds are: 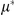 = −7.34, 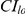 = −7.36, and 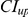 = −7.31. For women, the analogous estimates are: 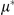 = −7.71, 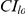 = −7.76, and 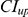 = −7.67. The estimates,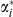, 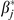, and 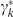, and their 95% 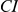 with the lower (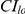) and upper (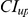) bounds are presented in Tables 6, 7, and 8, correspondingly. In these tables, the values of the anchored effects are presented in bold. In Table 5, the values of the identification parameters are presented in bold italic.

**Table 6:** Estimates of the Age effects, 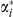 with the lower (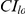) and upper (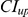) bounds of their 95% 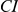, on LC occurrence in white men and women.

	Men			Women		
Age, *i*	α_i_^*^	*CI_lo_*	*CI_up_*	α_i_^*^	*CI_lo_*	*CI_up_*
**1**	−5.45	−5.64	−5.25	−4.46	−4.82	−4.11
**2**	−4.25	−4.40	−4.11	−3.38	−3.65	−3.11
**3**	−3.24	−3.36	−3.12	−2.52	−2.74	−2.30
**4**	−2.34	−2.44	−2.24	−1.77	−1.95	−1.59
**5**	−1.63	−1.71	−1.55	−1.24	−1.39	−1.09
**6**	−1.05	−1.11	−0.99	−0.78	−0.89	−0.67
**7**	−0.57	−0.61	−0.53	−0.43	−0.51	−0.35
**8**	−0.22	−0.24	−0.19	−0.16	−0.21	−0.11
**9**	**0**	----	----	**0**	----	----
**10**	0.10	0.07	0.12	0.03	−0.02	0.09
**11**	0.00	−0.04	0.05	−0.11	−0.20	−0.03
**12**	−0.36	−0.43	−0.30	−0.67	−0.79	−0.54

**Table 7:** Estimates of the TP effects, 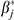, with the lower (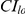) and upper (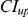) bounds of their 95% 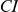, on LC occurrence in white men and women.

	Men			Women		
Time-period, *j*	β_j_^*^	*CI_lo_*	*CI_up_*	β_j_^*^	*CI_lo_*	*CI_up_*
**1**	0.50	0.42	0.59	−0.30	−0.46	−0.14
**2**	0.49	0.42	0.55	−0.13	−0.26	−0.01
**3**	0.42	0.37	0.46	−0.04	−0.13	0.05
**4**	0.28	0.25	0.32	0.00	−0.06	0.06
**5**	***0.14***	----	----	***0.03***	----	----
**6**	**0**	----	----	**0**	----	----

**Table 8:** Estimates of the BC effects, 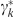, with the lower (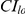) and upper (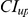) bounds of their 95% 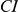, on LC occurrence in white men and women.

	Men			Women		
Birth-cohort, *k*	γ_k_^*^	*CI_lo_*	*CI_up_*	γ_k_^*^	*CI_lo_*	*CI_up_*
**1**	−0.83	−0.64	−1.01	−1.12	−1.52	−0.72
**2**	−0.64	−0.49	−0.78	−1.05	−1.34	−0.77
**3**	−0.46	−0.34	−0.58	−0.86	−1.09	−0.63
**4**	−0.33	−0.23	−0.42	−0.67	−0.85	−0.48
**5**	−0.24	−0.17	−0.32	−0.41	−0.56	−0.27
**6**	−0.17	−0.11	−0.22	−0.22	−0.33	−0.11
**7**	−0.13	−0.09	−0.17	−0.12	−0.20	−0.05
**8**	−0.03	0.00	−0.05	−0.04	−0.10	0.01
**9**	**0**	----	----	**0**	----	----
**10**	−0.05	−0.02	−0.08	−0.07	−0.12	−0.01
**11**	−0.11	−0.06	−0.15	−0.14	−0.23	−0.06
**12**	−0.23	−0.17	−0.30	−0.34	−0.46	−0.22
**13**	−0.35	−0.27	−0.43	−0.57	−0.72	−0.41
**14**	−0.37	−0.26	−0.48	−0.67	−0.86	−0.47
**15**	−0.36	−0.22	−0.50	−0.56	−0.81	−0.31
**16**	−0.39	−0.18	−0.60	−0.81	−1.18	−0.44
**17**	−0.47	−0.07	−0.88	−0.69	−1.31	−0.07

**Figure 1:**
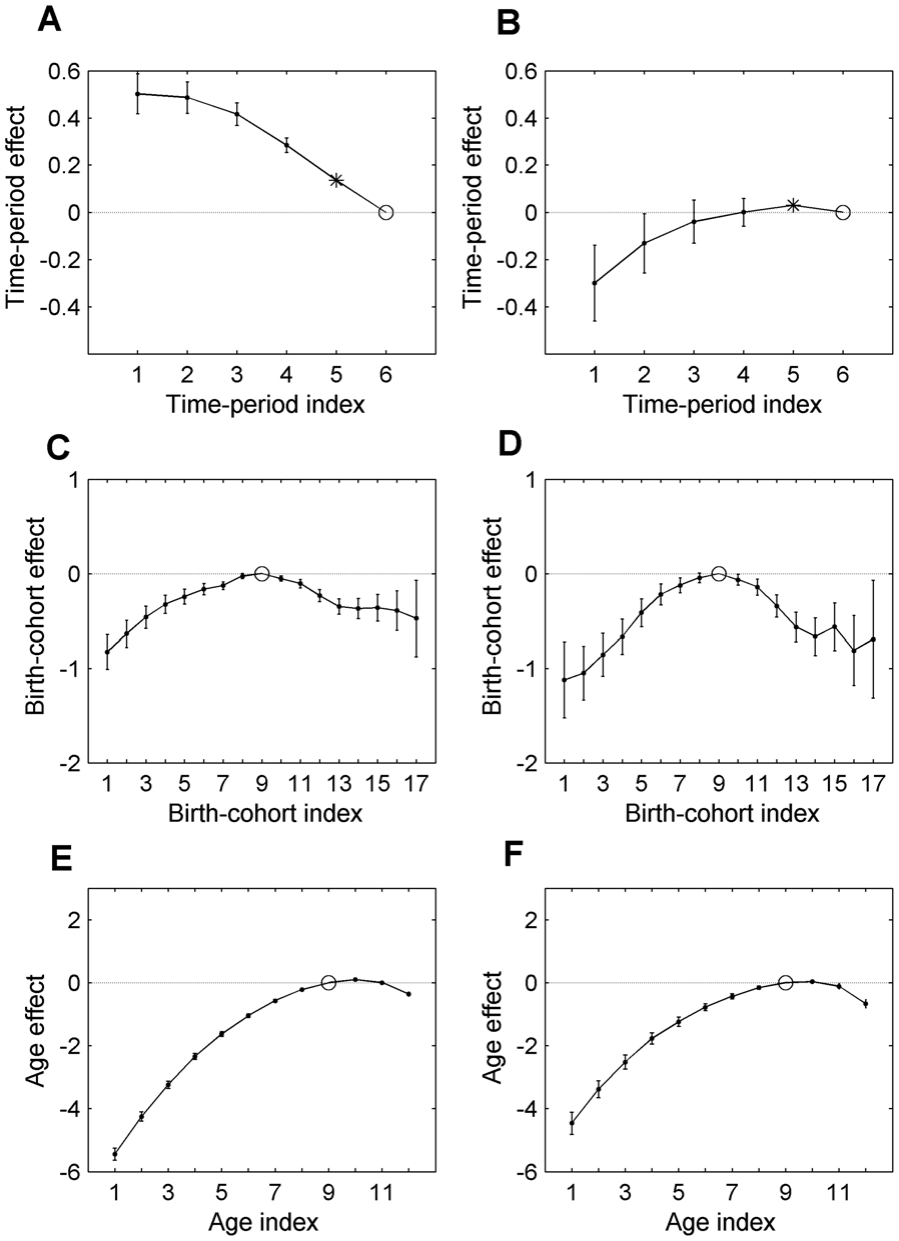
The Time-period (TP), Birth-cohort (BC) and Age effects on LC occurrence. Panels A and B present the trends of the TP effects for white men and women, correspondingly. Data are presented for six time periods (1975–79, 1980–94,…, 2000–04 years) indexed as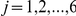. Panels C and D present the obtained trends of the BC effects for white men and women, correspondingly. Data are presented for 17 BC groups (1890–94, 1895–99,…, 1970–74 years) indexed as 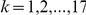. Panels E and F present the obtained trends of the Age effects *vs.* Age intervals (30–34, 35–39, …, 80–84, 85+ years), indexed as 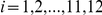, for white men and women, correspondingly. The anchored effects are presented by open circles. The identification parameters are presented by asterisks. The error bars show the 95% confidence intervals.

Figure 1 exhibits the results of the APC analysis of the LC occurrence in white men and women, anchored to the 2000–04 TP and to the 1930–34 BC. The anchored effects are presented by open circles. The identification parameters are presented by asterisks. The error bars show the 95% confidence intervals.

Panels 1A and 1B present trends of the TP effects on LC occurrence in white men and women, correspondingly. For men, these factors decreased from 1975 until 2004, while for women, these factors increased from 1975 to 1990 and then remained nearly constant.

Panels 1C and 1D present the obtained trends of the BC effects on LC occurrence in white men and women, correspondingly. For both men and women, these trends increase from the BC of 1890–94 until the BC of 1925–29, then decrease until the BC of 1950–54 and then remain almost unchanged.

Panels 1E and 1F present the obtained trends of the Age effects on LC occurrence in white men and women, correspondingly. These trends increase from Age 30 until Age 70–75 and, then, decrease at older ages.

**Figure 2:**
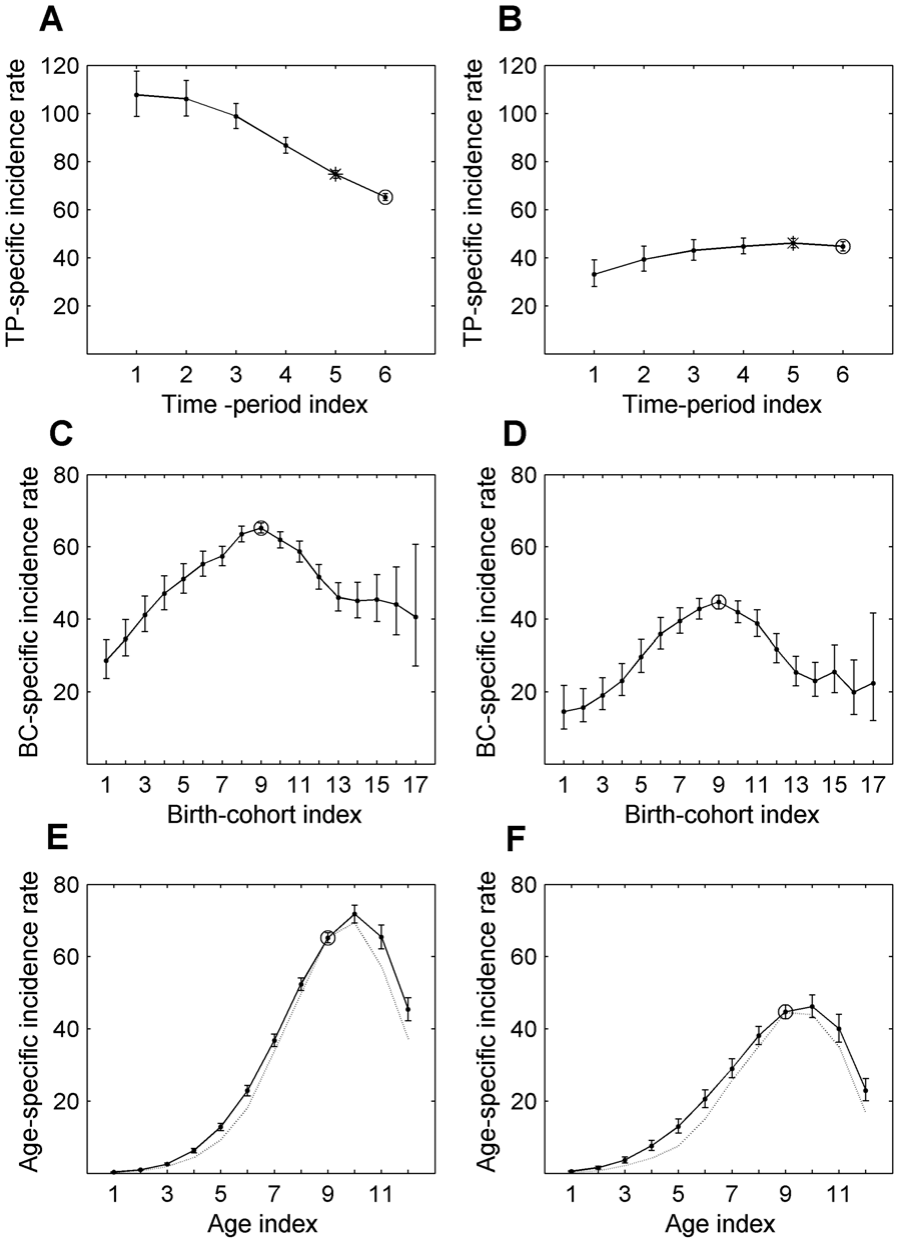
The TP-, BC- and Age-specific incidence rates of LC occurrence. Panels A and B present the TP-specific incidence rates in white men and women, correspondingly. Data are presented for six time periods (1975–79, 1980–94,…, 2000–04) indexed as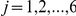. Panels C and D present the obtained BC-specific incidence rates in white men and women, correspondingly. Data are presented for 17 cohort groups (1890–94, 1895–99,…, 1970–74) indexed as 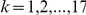. Panels E and F present the obtained Age-specific incidence rates *vs.* age intervals (30–34, 35–39, …, 80–84, 85+), indexed as 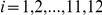, in white men and women, correspondingly. The cross-sectional Age-specific incidence rates, observed in the 2000–04 time period are shown by dotted lines. The anchored effects are presented by open circles. The error bars show the 95% confidence intervals.

Figure 2 demonstrates the APC effects on LC incidence rates in white men and women, anchored to the Age interval of 70–74, the TP of 2000–04, and the BC of 1930–34. The rates for the anchored Age, TP and BC are presented by open circles. The error bars show the 95% confidence intervals.

Panels A and B of this figure present the trends of the modeled TP-specific incidence rates *vs*. TP interval indexes,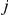, of LC in men and women, correspondingly. The estimates of the modeled TP-specific incidence rates, 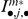, and their variances 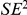 were obtained by formulas:
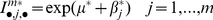
(12)

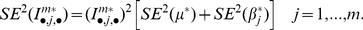
(13)For men, the TP-specific incidence rates of LC decreased from 1975 until 2004, while for women these increased from 1975 to 1990 and then remained nearly constant.

Panels C and D of Figure 2 present the trends of the modeled BC-specific incidence rates *vs*. BC interval indexes,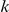, for men and women, correspondingly. The estimates of the modeled BC-specific incidence rates, 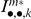, and their variances 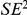 were obtained by formulas:
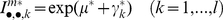
(14)

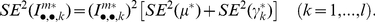
(15)For both men and women, the BC-specific incidence rates of LC increase from the cohort of 1890–94 until the cohort of 1925–29, decrease until the cohort of 1950–54 and then remain almost unchanged.

Panels E and F of Figure 2 present the cross-sectional Age-specific incidence rates, observed in the 2000–04 time period (dotted lines), and the estimates of the modeled Age-specific incidence rates anchored to the 2000–04 time period and to the 1930–34 birth cohort (solid lines) of LC in white men and women, correspondingly. The estimates of the modeled Age-specific incidence rates, 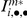, and their variances 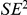 were obtained by formulas:
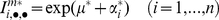
(16)

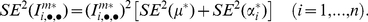
(17)The modeled Age-specific incidence rates at the anchored ages are shown by the open circles. The error bars show 95% confidence intervals. As can be seen, the modeled Age-specific incidence rates of LC in men and women have the “reverse bathtub” shapes that are increasing with Age, reaching maximum (at the age interval of 75–79) and then fall at older ages. It is important to notice that values of the modeled Age-specific incidence rates and the corresponding values of the observed cross-sectional Age-specific incidence rates are significantly different. This is because the estimates of the modeled Age-specific incidence rates are “cleaned-up” from the TP and BC effects, while the observed cross sectional Age-specific incidence rates are significantly influenced by these effects.

**Figure 3:**
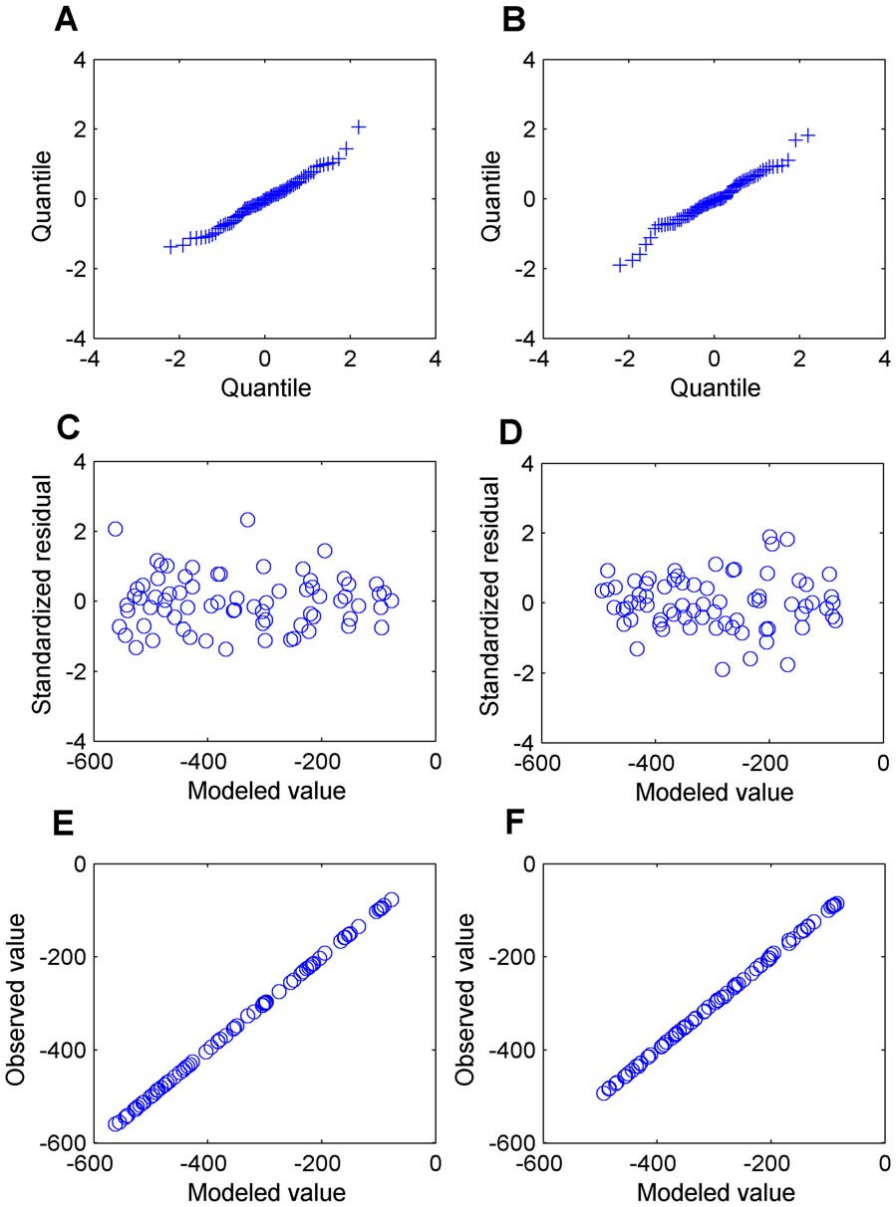
Validation of the performed model estimations of the APC effects on LC occurrences. Panels A and B exhibit the probability distribution plot of the standardized residuals, 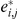, for white men and women, correspondingly. The vertical axes present the obtained quintiles of the standardized residuals and the horizontal axes show the corresponding quintiles of the standard normal distribution. The vertical axes of the panels C (for white men) and D (for white women) exhibit the standardized residuals, 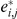, and the horizontal axes exhibit the modeled weighted values,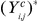. Panels E (for white men) and F (for white women) exhibit the observed weighted values, 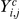, on the vertical axes *vs*. the modeled weighted values, 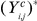, on the horizontal axes.

Figure 3 exhibits the results of assessing the validity of using the LLAPC model for determining the APC effects in the LC occurrences in white men and women. Panels 3A and 3B exhibit the probability distribution plot of the standardized residuals, 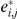. The vertical axes present the obtained quintiles of the standardized residuals and the horizontal axes show the corresponding quintiles of the standard normal distribution. For both men and women, the plots are sufficiently straight, except for several points which have very small or large quintiles.

The vertical axes of panels 3C and 3D exhibit the standardized residuals, 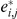, and the horizontal axes exhibit the modeled weighted values,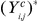. As seen from Panel 3C for men, all but two values of the standardized residuals, 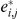, fall into the [−2,2] interval, while for women, all of these values are distributed within the interval. This indicates that the models of multiple regressions we used are appropriate for presenting the corresponding observational data.

Panels 3E and 3F exhibit the observed weighted values, 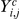, on the vertical axes *vs*. the modeled weighted values, 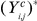, on the horizontal axes for men and women, correspondingly. For both men and women, the regression function used accurately models the actual observed values.

Overall, we can conclude that the LLAPC models used in this work fit the observational data of LC in white men and women.

## Discussion

For many decades, the problem of estimating the APC effects on cancer incidence rate data has intrigued researchers. The main difficulty in estimating these effects in the frame of the LLAPC model arises due to the fact that the APC effects are linearly interdependent temporal parameters and their values cannot be uniquely determined. Most of the known approaches for solving this identifiability problem have significant drawbacks and/or their computational implementation is complicated [2], [16].

In this work, we developed a new computationally effective approach for solving the identifiability problem in APC analyses. We showed that the solution of this problem can be reduced to a problem of determining one unknown identification parameter, 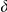.We used the effect, 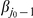, of the TP adjacent to the anchored TP,
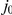
_,_ as such a parameter. We showed that when the identification parameter is *a priori* known, the identifiability problem with multiple estimators does not arise and a unique set of estimates of the APC effects can be found.

By using a heuristic assumption that the differences between the BC effects of the adjacent cohorts are close to 0, we showed that the optimal value of the unknown identification parameter can be obtained by minimizing (with respect to 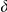) the weighted average of the squared differences between the adjacent BC effects. In other words, this procedure allows one to determine such a value of the identification parameter, which provides the “smoothest” trend within all possible trends of the BC effects. This heuristic assumption is milder than the one utilized in [17], where the use of smooth functions for presenting a temporal variation of the BC effects is required for assessing the APC effects. It should be noted that the aforementioned assumption was successfully used in our previous papers [18], [19].

In the present work, we extended the approach [2], [4], [8], [9] that is well-known as the “equate two effects” approach, in which all redundant parameters are equated to zero to solve the identifiability problem. Here, we used the LLAPC model with four redundant parameters to be identified. We equated one of the TP effects, one of the BC effects, and the corresponding Age effect to zero and used them as reference levels. We pointed out that by varying the fourth parameter, which we called the identification parameter, all possible solutions of the identifiability problem can be obtained. We proposed a method for obtaining the optimal value of the identification parameter, by which a unique set of the APC effects can be determined and thus the identifiability problem can be overcome.

We tested the proposed approach by estimating the APC effects on LC occurrence in white men and women. For this purpose, we used the Age-specific incidence rate data collected in the SEER 9 database during 1975–2004. By the aforementioned assumption and procedure, we determined the optimal values of the identifiability parameters and the corresponding unique sets of the APC effects on LC occurrence in white men and women.

We determined the modeled Age-specific incidence rates and showed that these rates have the “reverse bathtub” shape falling at old ages. This is consistent with several publications (see, for instance, [20]–[22]) suggesting the existence of a plateau, followed by a decline in the Age-specific cancer rates. In those studies, only the observational cross-sectional data were analyzed, while there was no accounting for the APC effects. In the present work, as well as in our previous studies [18], [19], we have shown that the curves presenting the modeled Age-specific cancer incidence rates also have the “reverse bathtub” shape when the APC effects are taken into consideration. At the present time, the vast majority of the existing Age-specific models of carcinogenesis (see [23]–[26] and references therein) are based on the assumption that cancer rates are increasing with age. There are only three models [27]–[29] that describe the “reverse bathtub” shape behavior of the Age-specific cancer rates. From these three models, the Weibull-like model [29] appears to have a better biological background.

Our analyses shows that the TP-specific incidence rates of LC in men decreased from 1975 until 2004, while in women, these rates increased from 1975 to 1990 and then remained nearly constant. Our results are consistent with the statement made in [30]: “…lung cancer incidence rates are declining in men and have leveled off after increasing for many decades in women. The lag in the temporal trend of lung cancer incidence rates in women compared to men reflects the historical difference in cigarette smoking between men and women; cigarette smoking in women peaked about 20 years later than in men.”

Our analysis also indicates that the variations of the BC-specific incidence rates of LC in men and women have similar shapes. This is a new result that was obtained by the approach presented in this work.

Overall, in our opinion, the present work provides the most efficient computational approach for determining the APC effects in the frame of the LLAPC model compared to other currently used approaches. The proposed approach can be used for the APC analysis of different types of cancer and other diseases as well.
